# Medical education during the COVID-19 pandemic: An online-only course can achieve good to medium psychomotor skill level of basic head and neck examination

**DOI:** 10.1016/j.heliyon.2024.e38044

**Published:** 2024-09-17

**Authors:** Axel Lechner, Stefan P. Haider, Pablo Escrihuela Branz, Benedikt Paul, Fatemeh Kashani, Martin Canis, Florian Schrötzlmair, Kariem Sharaf

**Affiliations:** Department of Otorhinolaryngology, University Hospital, LMU, Marchioninistr. 15, D-81377, Munich, Germany

**Keywords:** Medical education, Teaching of examination skills, COVID-19 pandemic, Online teaching, Otorhinolaryngology

## Abstract

**Background/Objectives:**

New teaching methods are warranted to meet the demand for increased flexibility in medical education while making optimal use of the limited resources of educators. The COVID-19 pandemic forced universities to resort to online-only teaching, even for training of psychomotor skills. The objectives of this study were: (I) to investigate the performance of students without previous experience in ear, nose and throat (ENT) examination after completing an asynchronous online teaching course in an objective standardized clinical examination (OSCE) and (II) to evaluate the degree of over- and underestimation of their abilities.

**Methods:**

The study was designed as a prospective, single-institution medical education cohort study. Medical students (n = 54; 3rd/4th year, no previous head and neck examination skills) completed a comprehensive online training for basic head and neck examination skills 1–14 days before undergoing an OSCE on 5 different examination items (cervical lymph node examination, oral/oropharyngeal examination, otoscopy, Weber/Rinne hearing test, anterior rhinoscopy). Expert-evaluated theoretical knowledge and practical abilities were measured using Likert scales (1 = high proficiency to 5 = low proficiency). Participants self-evaluated their own skills before and after the OSCE (5-point Likert scales).

**Results:**

Students achieved an overall good to medium skill level in basic head and neck examination after online training. Increased psychomotor complexity of tasks contributed to worse performance. Expert evaluation of practical skills was better in male participants and those who rated their preparation as thorough. Low/no misjudgment (discrepancy between self- and expert-evaluation) was observed in most participants, while high discrepancy levels (strong misjudgment) were mainly attributable to the students’ underestimation of their own abilities.

**Conclusions:**

Online training of head and neck examination skills is a cost- and resource-efficient introduction to skill training and was inevitable during the pandemic. However, it cannot replace practical hands-on training, especially in tasks with high psychomotor requirements. Refresher-courses could compensate for cancelled skill training during the pandemic.

## 1 Introduction

Increasing digitalization has changed the way of teaching in medical schools already in the years before the onset of the COVID-19 pandemic [1]. Contact restrictions during the COVID-19 pandemic served as a major catalyst for this development, as universities were forced to adapt their curricula to the new circumstances within weeks or months [2]. Lectures and seminars could be readily transferred online and suitable software solutions created the basis for interactive e-learning concepts. Temporary suspension or reduction of in-hospital teaching required to train students in clinical skills in an online-dominated setting. Providing adequate teaching by online distance learning as substitute for hands-on examination courses or surgical skill trainings posed a major challenge for medical schools [3]. Given the fact that head and neck examination involves the upper airways with a high risk of SARS-CoV2 virus transmission during examination, the impact of restrictions was exceptionally high for ear, nose and throat (ENT) training [4]. In the first months of the pandemic, substantial lack of personal protective equipment aggravated the situation. Concepts such as supervised social bubbles of few students [5], remote surgical skill training with subsequent feedback [6,7] or unsupervised online-only training of practical procedures [8] emerged in most specialties including ENT.

The effectiveness of supervised online distance education in ENT was evaluated by few studies. For example, ENT examination gradual skill improvement after multiple repetitions was noted in a supervised online course [9], emphasizing the importance of practical execution of practical tasks [10]. However, the effectiveness of unsupervised substitute concepts regarding development of psychomotor skills and retaining them in the long-term remains largely unclear.

A fundamental capability of medical students and doctors besides actual skill competence should be the ability of appropriate self-assessment [11]. A significant mismatch between perceived and actual competence, i.e. under-/overestimation of one's proficiency can lead to misdiagnosis and put patients at risk. In previous studies, we could show that experienced students are mostly able to assess their own competencies in specialized ENT examination adequately after completing online or in-hospital training. Misjudgment was mainly attributable to underestimation of the students' own skills [12,13].

While it was inevitable to conduct online courses during the pandemic, the impact of reduced face-to-face training and suspension of clerkships on students’ competencies especially in practical clinical skills, is not fully established yet.

Hence, this study aimed at investigating self- and expert-assessed competencies of students after they completed a comprehensive head and neck examination online training. Participating students were in early clinical training and had no prior practical ENT experience. ENT examination skills that are relevant to different specialties such as general practitioners or pediatricians were the scope of the training.

## 2 Material and methods

### 2.1 Teaching of examination skills in the medical education curriculum (before/during COVID-19 pandemic)

Within the medical school curriculum (MeCuM) at Ludwig-Maximilians-University (LMU), students get in contact with patients as early as in 2nd year of university, starting with fundamental examination training (cardiopulmonary and abdominal examination). Basic head and neck examination skills are taught during 3rd/4th year and ENT-specific examination during 5th year. However, due to restrictions during the COVID-19 pandemic, students did not receive hands-on teaching of ENT examination skills. We therefore introduced online courses to not only provide lectures and seminars, but also teach practical examination skills.

### 2.2 Course structure

**Fig. 1:**
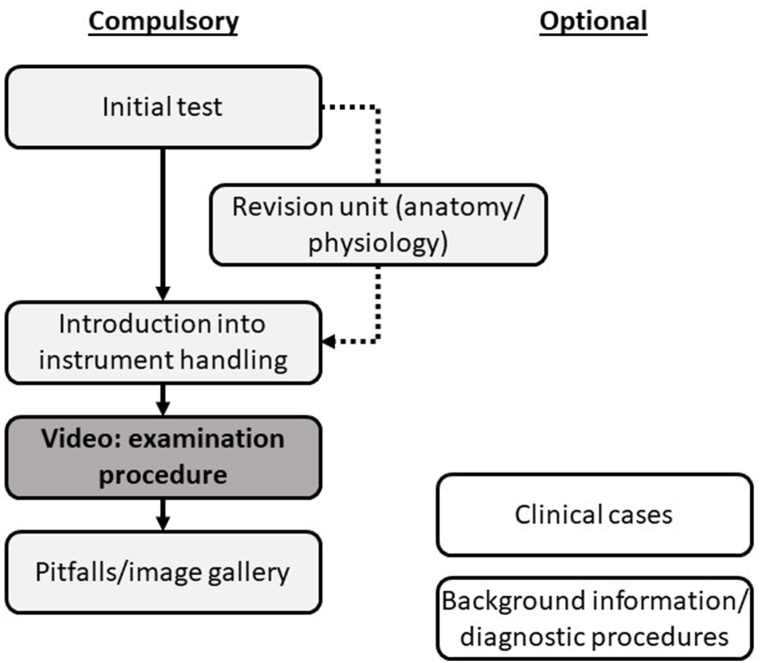
**Structure of the online course** Compulsory content is centered around detailed examination videos (left), optional content includes clinical cases, background information and e.g. possible diagnostic procedures following physical examination.

The aim of the online course that was developed from May 2018 to February 2020 was to comprehensively prepare for basic head and neck examination, offering compulsory and optional content (Fig. 1). Based on the results of a short initial test, students could complete a revision unit on anatomy and physiology or continue with the actual course. The next subunit of the course covers theoretical principles of the examination and necessary instruments. Afterwards, a detailed training video demonstrates the complete examination item, followed by additional information on pitfalls and image galleries of examination findings (normal/pathological). Optional course content comprises video podcasts with anatomy/physiology background and clinical cases applying basic head and neck examination. A short examination video for revision purposes is also included.

The following examination items are covered in the structure outlined above: (1) cervical lymph node examination, (2) oral/oropharyngeal examination, (3) otoscopy, (4) Weber/Rinne hearing test and (5) anterior rhinoscopy.

Focusing only on the examination videos and image galleries, the estimated time to complete the course is about 3–4 h. Completing the whole course materials to use them for extensive learning and answering a written exam about the examination techniques is supposed to take about 24 h in total.

### 2.3 Participants

Medical students (3rd/4th year) completed the online course, followed by an objective standardized clinical exam (OSCE) 1–14 days after the course. Notably, all participating students did not have any prior head and neck examination experience irrespective of their year in medical school. Eligible students (n = 149; female n = 110; male n = 39) received an email notification regarding the study 14 days before the date of the exam, participation was voluntary and in compliance with governmental and hospital COVID-19 safety measures. Every participant underwent COVID-19 PCR testing the day before the OSCE. Students, who did not complete the online course or already had head and neck examination experience e.g. from a clinical elective, were excluded from the analysis (n = 2).

### 2.4 Objective structured clinical exam

Participants fulfilling the inclusion criteria for the study completed a questionnaire assessing basic biographic data (age, sex, semester count), online course use (duration, timepoint, self-assessed diligence of preparation) and self-assessed level of general examination skills and estimated skill level in aforementioned five head and neck examination items. Likert scales ranging from 1 (high proficiency) to 5 (low proficiency) were used for assessment. The OSCE started with written instructions for every examination item covering theoretical knowledge and practical execution. Theoretical, practical and overall performance was measured by one of two experienced medical teachers separately (A.L. and K.S.) in a standardized way using the same Likert scales as in the questionnaire. Both teachers share 4 years of joint teaching of head and neck examination and aligned expectancy levels before the study to ensure a high inter-rater concordance. The degree of proficiency was aligned in a way that the Likert scale were able to discriminate relevant differences and a ‘3’ reflecting a mediocre performance based on the estimated clinical knowledge/skill regarding the students' advance in medical studies (main goal of examination achieved). High proficiency (‘1’) was achieved when all relevant aspects of the examination were covered, e.g. in the oral/oropharyngeal examination, all anatomical subsites were inspected to allow for detection of potential abnormal findings. In low proficiency (‘5’) performance, even basic principles of the examination were missing, e.g. incorrect instrument handling.

After the OSCE and structured feedback, participants reevaluated their skill level Likert scale-based again.

The study was conducted based on the approval by the ethics committee of the local medical faculty (Ethikkommission der Medizinischen Fakultät der Ludwig-Maximilians-Universität, IRB approval number 19–333) and in compliance with the WMA Declaration of Helsinki.

### 2.5 Data analysis and statistical analysis

Misjudgment and over-/underestimation were calculated by taking the Likert scale point difference between self-assessment and expert-assessment. Misjudgment indicates the overall level of non-concordance of self-assessment and expert-evaluation: Δ(│expert-evaluation - self-assessment│). Over-/underestimation is calculated by Δ(expert-evaluation - self-assessment), thus additionally taking into account the direction of misjudgment. Positive values indicate that students overestimate their own skill level, negative values show underestimation [12].

Statistical analysis was performed with GraphPad Prism 9 (San Diego, CA, USA). Paired or unpaired student's t-test were used for comparison of two paired or unpaired groups, while ANOVA was used for comparison of more than two mean values in concordance with previous publications regarding the statistical analysis of data obtained from Likert scales [14,15]. Graphs were generated using GraphPad Prism 9. Numbers in the results section, figures and tables are shown as mean ± respective standard deviation (mean ± SD).

## 3 Results

In total, n = 54 students without previous head and neck examination training participated voluntarily in the study (36.2 % of all eligible students). Student characteristics are summarized in Table 1. Participants were predominantly female (77.8 %) and on average moderately interested in ENT, which was comparable to previous studies before/during the COVID-19 pandemic at our institution [12,13]. 44 participants were in 3rd year of medical school, 10 students in 4th year. Most participants completed the online course within 3 days before the OSCE (41.9 %), while 35.5 % did so 7–14 days prior to the exam. Self-evaluated general examination skills were rated 2.37 ± 0.71 (mean ± SD; 1 = high proficiency to 5 = low proficiency; Table 1).

**Table 1:** Student characteristics (data shown as mean ± standard deviation).

Number of students		n = 54
Age		24 ± 3.5 years
Gender	Female	42 (77.8 %)
	Male	11 (20.4 %)
	n.a.	1 (1.8 %)
Semester		6.4 ± 1.1
Interest in ENT (1 = very high to 5 = very low)		2.68 ± 0.92
Self-assessment of general physical examination skills (1 = high proficiency to 5 = low proficiency)		2.37 ± 0.71
Timepoint of online course completion	1–3 days prior to exam	39 (72.2 %)
	4–7 days prior to exam	7 (13.0 %)
	>7 days prior to exam	8 (14.8 %)

### 3.1 Online-only practical training can achieve good to medium basic head and neck examination skills in students without ENT experience

**Fig. 2:**
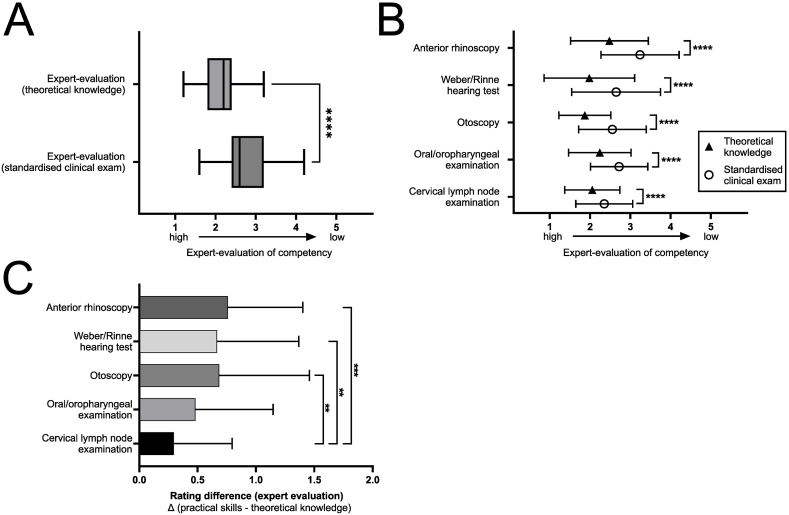
**Expert-evaluation of practical skills vs. theoretical knowledge.** Performance in theoretical knowledge and practical performance is compared (A) overall and (B) in different examination items. Rating differences between practical skill performance and theoretical knowledge are shown for every examination item (C). Evaluation is measured on a 5-point interval scale (Likert scale; 1 = high proficiency to 5 = low proficiency); ∗∗p < 0.01, ∗∗∗p < 0.001, ∗∗∗∗p < 0.0001.

Performance during the OSCE was evaluated separately for theoretical knowledge and practical execution of every examination item. Students achieved good levels of overall theoretical knowledge (2.13 ± 0.49), which was significantly better than their practical performance (p < 0.0001). However, their practical skills were still rated good to medium on average (2.70 ± 0.56; Fig. 2A). While expert evaluation of theoretical knowledge was comparable for all five examination items, results for practical execution showed greater heterogeneity. Significant differences between theoretical and practical performance were pervasive across all five examination items (all p < 0.0001, Fig. 2B). Expert-evaluated execution of cervical lymph node examination was rated best (2.35 ± 0.70) and anterior rhinoscopy results worst (3.24 ± 0.97). The point difference between theoretical and practical performance was the lowest in cervical lymph node examination (0.30 ± 0.50), being significantly lower than in Weber/Rinne hearing test (0.67 ± 0.70; p = 0.0044; Fig. 2C) and in tasks with the highest requirement of psychomotor skills, namely otoscopy and anterior rhinoscopy (0.69 ± 0.77; p = 0.010 and 0.76 ± 0.64; p = 0.0003, respectively; Fig. 2C).

### 3.2 An in-depth online preparation makes a difference in practical skills but not in theoretical knowledge

**Fig. 3:**
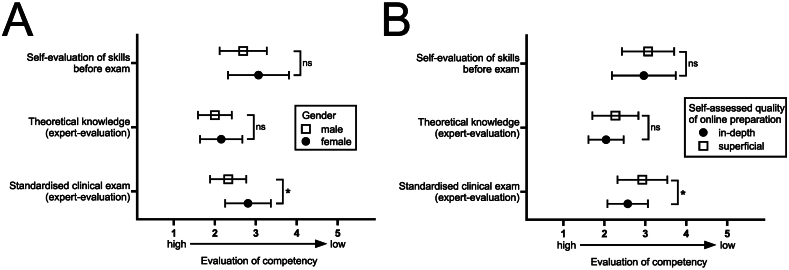
**Differences in OSCE results in selected subgroups (gender, self-assessed thoroughness of preparation).** Results of self-evaluation, expert-evaluated theoretical knowledge and practical performance are depicted in separate graphs for subgroups (A) female/male and (B) for self-assessed profoundness of online-course preparation. (Likert scale; 1 = high proficiency to 5 = low proficiency); ns = not significant, ∗p < 0.05.

Subsequent subgroup analyses according to age, interest in ENT, timepoint of online course completion (subgroups: completion 1–3 vs. 4–6 vs. 7–14 days before the exam) or self-assessed general examination competency did not show any differences regarding expert-evaluated skill levels and theoretical knowledge (data not shown). Students who stated that they gained at least sufficient practical skills after completing the online-only training (rating 1–3 on 5-point Likert scale), consequently rated their own skills higher than students who did not agree that online-only teaching provides sufficient training (2.74 ± 0.71 vs. 3.20 ± 0.69, p = 0.020). However, both groups performed similar in the theoretical and practical expert evaluation (2.11 ± .0.45 vs. 2.14 ± 0.53 and 2.64 ± 0.48 vs. 2.75 ± 0.62, p = ns). While we did observe a significant difference in expert-evaluation of practical skills according to gender (2.33 ± 0.44 (male) vs. 2.81 ± 0.56 (female), p = 0.011; Fig. 3A), respective differences in theoretical knowledge and self-evaluation of skills were insignificant. A similar pattern was observed when subdividing participant groups according to their preparation for the OSCE. Students, who rated their preparation as thorough, performed significantly better in practical skills (2.57 ± 0.50 vs. 2.93 ± 0.61, p = 0.022). Yet, their theoretical knowledge and self-assessment of skills was not significantly better as in students rating their preparation as less diligent (Fig. 3B).

### 3.3 Majority of students self-assesses practical skill level with high accuracy

**Fig. 4:**
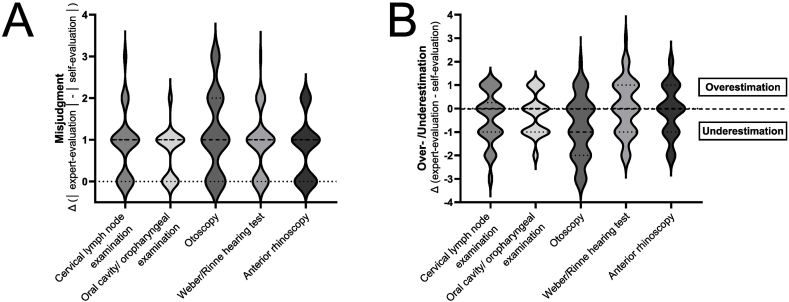
**Individual differences between expert-evaluation and self-evaluation in different examination items (misjudgment).** Misjudgment of one's abilities calculated as difference between expert-evaluated practical execution and upfront self-evaluation irrespective of direction is shown in violin plots for every examination item (A). Violin plots demonstrate direction and degree of misjudgment (over-/underestimation) in every item (B).

An important capability in medicine is to know one's limits and competencies. In particular, self-assessment of practical tasks that require a certain level of psychomotor skills has to be adequate. Misjudgment of one's skills can undoubtedly result harming a patient or in unreliable results of the examination. If the required task has only been taught in theory but never been executed before, reasonable self-assessment skills and self-reflection are even more indispensable. We therefore assessed the ability of participants to assess their level of competency before the OSCE. Misjudgment and over-/underestimation in self-assessment compared to expert-evaluation were calculated as outlined above. Differences between expert- and self-evaluation of 0–1 on Likert-scales were labelled no/minimal misjudgment, differences >1 as strong over-/underestimation. Overall, the majority of students was able to assess their abilities adequately. No/minimal misjudgment was observed in 81.9 % examinations and conversely in 18.1 % strong misjudgment (Fig. 4A). In detail, rates of minimal/no misjudgment were 66.7 % (otoscopy), 81.5 % (Weber/Rinne hearing test), 83.3 % (cervical lymph node examination and anterior rhinoscopy) and 94.4 % (oral cavity/oropharyngeal examination). Even though psychomotor skill requirements for otoscopy and rhinoscopy are comparably high, respective strong misjudgment rates were 33.3 % and 16.7 %, largely dominated by underestimation in otoscopy (Fig. 4B). In general, no clear link between skill requirements or expert-evaluation and level of misjudgment could be demonstrated. Underestimation of one's skills was predominant in all examination items except for Weber/Rinne hearing test, where over- and underestimation was balanced (Fig. 4B).

### 3.4 Strong improvement of self-evaluated skills after OSCE/training and structured feedback

**Fig. 5:**
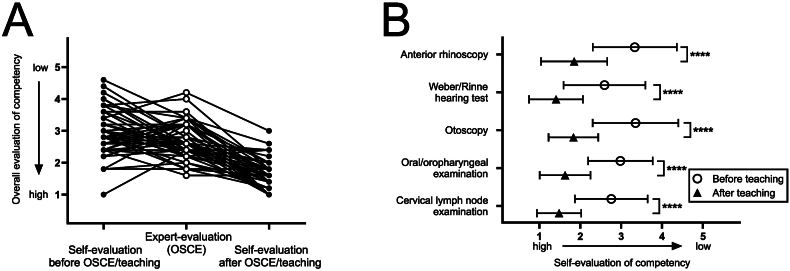
**Comparison of overall self-evaluation before and after OSCE and structured feedback.** Changes in individual overall self-evaluation are shown before and after OSCE compared to expert-evaluated practical skills (A). Self-evaluated skills for every single examination item are depicted (B). Evaluation is measured on a 5-point Likert scale (1 = high proficiency to 5 = low proficiency); ∗∗∗∗p < 0.0001.

The main goal of this project was to assess online-only training. However, we additionally compared self-evaluated competencies before and after completing the OSCE and having received structured feedback. As expected, almost all participants indicated improvement of their own skill level with higher homogeneity of data, indicating a certain level of alignment after intervention (3.00 ± 0.73 vs. 1.64 ± 0.47, p < 0.0001; Fig. 5A). Distribution of expert-evaluated practical skills was comparable to self-evaluation before the OSCE, ranging from 1.6 to 4.2, while self-evaluated skills after the OSCE ranged from 1.0 to 3.0 (Fig. 5A). Every examination item was self-evaluated at least one figure higher on the 5-point Likert scale than before the OSCE/teaching with highest improvement rates for examination items with high psychomotor requirements (otoscopy (1.52 ± 1.19) and anterior rhinoscopy (1.48 ± 1.21); Fig. 5B).

### 3.5 What is the student's opinion on online-only teaching of head and neck examination skills?

**Fig. 6:**
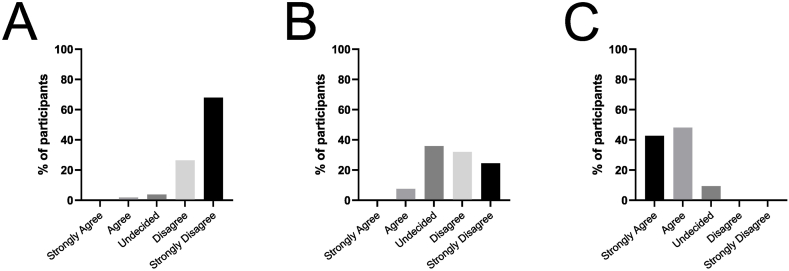
**Student's opinion on online-only teaching (Likert scale-based survey).** Percentages of answers are shown in bar diagrams on following questions (before OSCE): (A) Could this online teaching completely replace practical teaching lessons? (B) Did you gain sufficient knowledge in practical head and neck examination through this course? (C) The only course is a motivating experience for subsequent practical training.

We asked the participants whether the online course could replace curricular head and neck examination training. 94 % of students (strongly) disagreed with this notion (Fig. 6A). However, only 56 % of students (strongly) disagreed that they personally gained sufficient practical clinical head and neck examination skills by completing the online course while 36 % were undecided and 8 % agreed (Fig. 6B). Almost all participants valued the online course as a motivating preparation for hands-on training (Fig. 6C).

## 4 Discussion

The COVID-19 pandemic led to unprecedented rapid changes in curricular medical education and to the development of innovative teaching concepts. However, the impact of these changes on knowledge and skill level of students could often not be assessed. To our knowledge, this is the first study investigating the potential of online-only teaching of basic head and neck examination skills in unexperienced students, reporting skill levels and discrepancy of expert- and self-evaluation. Major limitations of the study include the total number of participants (n = 54) and the low number of male students (n = 12; 22,2 %), therefore limiting its generalizability. Additionally, we cannot provide follow-up data regarding long-term skill retention and expert-evaluation based on 5-point Likert scales does not fully prevent subjective influence on ratings. However, this study contributes to the still scarce knowledge regarding psychomotor skill development in online-only courses.

In many medical schools including ours, face-to-face courses were replaced by online teaching formats due to contact restrictions. Although participating students had no prior practical experience, their basic head and neck examination skills after completing the online training was rated as good to medium, which is remarkable given the fact that the examination requires a high level of psychomotor coordination. Other studies reported high levels of competence in head and neck examination in online hands-on learning concepts already after short training intervals [9,10]. A study in dental students reported comparable skills in the application of local anesthesia between face-to-face, blended and online-only teaching groups [16] and e-learning was implemented to teach basic surgical skills and psychomotor skills in nursing, demonstrating satisfying skill levels and improved confidence [7,17]. Interestingly, online courses resulted in better practical cardiopulmonary examination skills compared to face-to-face courses in another study [18]. However, online-only head and neck examination courses have not been evaluated so far to our knowledge.

We saw a rapid improvement of skill confidence after practical execution of the examination and feedback and additionally a strong alignment of skill levels. This observation emphasizes the importance of practical training and individual feedback, preferably within an inverted-classroom setting. However, a substitute for this resource-intensive concept might be training at home with disposable instruments. Lakner et al. reported preliminary results with higher scores in video-based evaluation of head and neck examination when students received teleteaching with instruments at home compared to training without instruction [19]. E-learning courses with (un-) supervised training could also serve as a time-efficient concepts to refresh clinical skills. ENT as a specialty with limited exposure during medical training might also benefit from introducing new teaching methods developed during the pandemic, such as virtual electives or near-peer teaching, which in turn can improve the students’ confidence in head and neck examination competencies [[20], [21], [22], [23]].

It is not surprising that students in our study, who prepared thoroughly for the course, showed better practical performance. However, differences of practical performance according to gender should be interpreted with caution due to the low percentage of participating male students (n = 12). Male and female students’ self-evaluation was comparable, which is consistent with other studies on medical students [24,25]. While there is still insufficient knowledge on gender differences in medical education, a recent study could demonstrate higher rates of anxiety in female medical students compared to male students, negatively impacting their performance [26]. However, another study reported better performance of female medical students irrespective of the teaching method (face-to-face/online learning) [27].

Realistic self-assessment is a crucial quality every medical student must develop during medical school. Misjudgment of one's skills will eventually jeopardize a patient's health and safety. The acquisition of accurate self-reflection and -assessment is a complex process influenced by different factors such as own experience, observation of others, social interaction or feedback [11,28]. While it was shown that self-reflection skills improve by age and semester [11], it could also be shown, that the lowest performers showed the highest inaccuracy in self-assessment already before the pandemic [29]. By drastically reducing in-hospital training, students were restricted in developing self-reflection and -assessment during the pandemic. We could recently show that experienced students performing specialized ENT examination showed higher rates of misjudgment when they completed an online-only examination course compared to traditional hands-on training [12]. Here, we evaluated basic head and neck examination skills, observing encouragingly high concordance of self- and expert evaluation irrespective of psychomotor difficulty of the respective examination. Misjudgment was mainly due to underestimation of one's skills, which is in line with previous findings [25,30]. Interestingly, Inoue et al. reported lower misjudgment in dental students during the pandemic despite less face-to-face training [30]. However, the long-term impact of the pandemic on the accuracy of self-assessment of practical skills among medical students and recent graduates remains to be established.

Although students value the flexibility of online courses and the low barriers to get insights into specialties such as ENT, they do not see distance learning as an adequate replacement of in-hospital training [21,[31], [32], [33], [34]]. They understand the necessity of distant learning and increased self-directed learning, but also demand a more interactive learning environment and patient contact [2,35]. Participants in our study appreciated the online course as a motivating preparation, but not as a substitute for hands-on training. In our experience, the incorporation of online courses as part of blended-learning in practical skill training in our medical school curriculum after the pandemic is well-accepted by students, resource-effective and contributes to standardization of medical education. They could furthermore help in long-term retention of skills as they can be easily accessed for individual practice. E-learning tools in practical skill training should be seen as an inevitable last resort as stand-alone courses during the pandemic and their valuable addition to face-to-face teaching is undeniable.

## 5 Conclusion

This study showed that online-only teaching of practical basic head and neck examination skills can achieve good to medium results in medical students without prior ENT exposure. The overall level of misjudgment of skills was relatively low among participants. Misjudgment was predominantly caused by underestimation of one's skills. However, the long-term effect of online-only vs. thorough face-to-face training on practical examination skills needs to be established in future studies. Refresher training of practical skills for the ‘pandemic generation’ of medical students are therefore warranted to ensure a satisfactory level of practical skills for their future medical career.

## Funding

Development and implementation of the online examination course was funded by the Bavarian Virtual University (Virtuelle Hochschule Bayern; vhb). The funding source was not involved in study design, collection, analysis and interpretation of data or writing of the report.

## Ethics approval

Data sampling, data processing and analysis were approved by the ethics committee of the local medical faculty (Ethikkommission der Medizinischen Fakultät der Ludwig-Maximilians-Universität, IRB approval number 19–333, 06/2019) and were in compliance with the WMA Declaration of Helsinki. Written informed consent was obtained from participants.

## Availability of data and material

Data and detailed statistical analyses of this study are available from the corresponding authors upon request.

## CRediT authorship contribution statement

**Axel Lechner:** Writing – review & editing, Writing – original draft, Project administration, Funding acquisition, Data curation, Conceptualization. **Stefan P. Haider:** Investigation, Conceptualization. **Pablo Escrihuela Branz:** Data curation. **Benedikt Paul:** Writing – review & editing, Visualization, Investigation, Data curation, Conceptualization. **Fatemeh Kashani:** Writing – review & editing. **Martin Canis:** Writing – review & editing. **Florian Schrötzlmair:** Writing – review & editing, Supervision. **Kariem Sharaf:** Writing – review & editing, Writing – original draft, Supervision, Investigation, Funding acquisition, Data curation, Conceptualization.

## Declaration of competing interest

The authors declare that they have no known competing financial interests or personal relationships that could have appeared to influence the work reported in this paper.
